# The American Illustrated Medical Dictionary

**Published:** 1901-10

**Authors:** 


THE AMERICAN ILLUSTRATED MEDICAL DICTIONARY. A new and complete dictionary of the terms used in medicine, surgery, dentistry, pharmacy, chemistry and the kindred branches, with their pronunciation, derivation and definition. Including much collateral information of an encyclopedic character. By W. A. Newman Dorland, A. M., M. D., Assistant Obstetrician to the University of Pennsylvania Hospital; editor of the American Pocket Medical Dictionary, etc. Together with new elaborate tables of arteries, muscles, nerves, veins, etc.; of bacilli, bacteria, diplococci, micrococci, streptococci, ptomaines and leukomains; weights and measures; eponymic tables of diseases, operations, signs and symptoms, stains, tests, methods of treatment, etc.; with numerous illustrations and twenty-four colored plates. Pp., 770; price, $4.50; with index, $5.00. Philadelphia and London: W. B. Saunders & Co. 1901.

This book is the correct size for general use. The back is leather and flexible, and the type is clear and print in double column. The preface in short. The book shows for itself. T. J. B.

